# The Impact of the *IKBKG* Gene on the Appearance of the Corpus Callosum Abnormalities in Incontinentia Pigmenti

**DOI:** 10.3390/diagnostics13071300

**Published:** 2023-03-30

**Authors:** Snežana Minić, Nataša Cerovac, Ivana Novaković, Slobodan Gazikalović, Svetlana Popadić, Dušan Trpinac

**Affiliations:** 1 Clinic of Dermatovenerology, University Clinical Center of Serbia, Faculty of Medicine, University of Belgrade, Deligradska 34, 11000 Belgrade, Serbia; prof.svetlana.popadic@gmail.com; 2 Clinic for Neurology and Psychiatry for Children and Youth, University Clinical Center of Serbia, Dr. Subotica 6a, Faculty of Medicine, University of Belgrade, 11000 Belgrade, Serbia; natasa.cerovac.npk@gmail.com; 3 Institute of Human Genetics, Faculty of Medicine, University of Belgrade, Višegradska 26, 11000 Belgrade, Serbia; tetaana61@yahoo.com; 4 Institute for Mother and Child Healthcare of Serbia “Dr Vukan Čupić”, Radoja Dakića 8, 11070 Belgrade, Serbia; progazi@gmail.com; 5 Institute of Histology and Embryology, Faculty of Medicine, University of Belgrade, Višegradska 26, 11000 Belgrade, Serbia; dusantrpinac@gmail.com

**Keywords:** incontinentia pigmenti, *IKBKG* gene mutation, central nervous system (CNS), corpus callosum, whole exome sequencing (WES), X-chromosome inactivation, magnetic resonance imaging (MRI)

## Abstract

Incontinentia pigmenti (IP) is a rare skin disease combined with anomalies of the teeth, eyes, and central nervous system (CNS). Mutations of the *IKBKG* gene are responsible for IP. Among the most frequent CNS abnormalities found in IP using magnetic resonance imaging (MRI) are corpus callosum (CC) abnormalities. The aim of the study was to determine the presence of CC abnormalities, their relationship with the *IKBKG* mutations, and the possible presence of mutations of other genes. A group of seven IP patients was examined. Analyses of the *IKBKG* gene and the X-chromosome inactivation pattern were performed, as well as MRI and whole exome sequencing (WES) with the focus on the genes relevant for neurodegeneration. WES analysis showed *IKBKG* mutation in all examined patients. A patient who had a mutation of a gene other than *IKBKG* was excluded from further study. Four of the seven patients had clinically diagnosed CNS anomalies; two out of four had MRI-diagnosed CC anomalies. The simultaneous presence of *IKBKG* mutation and CC abnormalities and the absence of other mutations indicate that *IKBKG* may be the cause of CC abnormalities and should be included in the list of genes responsible for CC abnormalities.

## 1. Introduction

Incontinentia pigmenti (IP [MIM 308300], Bloch–Sulzberger syndrome, ORPHA464) is a rare X-linked genodermatosis with an estimated birth prevalence of 1.2/100,000 [1] in which changes in skin and skin appendages are usually combined with anomalies of other organs, teeth, eyes, and central nervous system (CNS) [2]. IP appears almost exclusively in females and is usually lethal in males [3,4]. Mutations of the *inhibitor of kappaB kinase gamma* (*IKBKG,* previously *NEMO*) gene, localized on the X-chromosome, locus Xq28, are responsible for IP [5]. Skewed X-chromosome inactivation, caused by counter-selection of cells expressing the mutation, is often observed [6]. *IKBKG* gene product activates nuclear factor-kappa B (NF-κB), a transcription factor that regulates the expression of hundreds of genes in almost all cells and is involved in cell proliferation, cell survival, the cellular stress response, innate immunity and inflammation [7]. The highest expression level of *IKBKG* was noted in the CNS [8]. According to a systematic review of CNS anomalies in IP performed on 1393 IP patients, the most frequent types of CNS anomalies were seizures, motor impairment, mental retardation, and microcephaly, of 41.98%, 25.70%, and 20.36%, respectively [9]. The most frequently registered CNS lesions found using brain imaging methods were brain infarcts or necrosis, brain atrophies, and corpus callosum (CC) lesions, of 24.55%, 17.36%, and 13.17%, respectively [9]. The significant occurrence of CNS anomalies in IP patients led to their inclusion in the updated IP diagnostic criteria [10]. In a group of 44 examined IP patients with neurological disorders who underwent magnetic resonance imaging (MRI), one patient had agenesis, and eight had hypoplasia of the CC. In all nine patients, CC abnormalities were associated with other CNS disorders [11].

The CC is the major neural pathway that connects the left and right cerebral hemispheres [12]. It is traditionally divided into four segments, the rostrum, genu, body, and splenium [13]. The first axons cross that callosal plate at 74 days of embryonic development, initially forming the CC as a small bundle that expands greatly to form a broad band connecting a large part of the base of the cerebral hemispheres [14,15] (p. 232). CC formation is complete by 115 days [15] (p. 232), and its formation is mediated by many genes [16]. Axons for the CC are generated from the small pyramidal neurons of layer 3 of the mature cortex [14]. The CC involves axons from a widespread area of cerebral neocortex that includes frontal, parietal, temporal and occipital lobes [14]. Interhemispheric transfer of information is the principal understood function of CC, but it also synchronizes the two cerebral hemispheres in terms of electrophysiological activity [14].

CC development depends on a large number of different cellular and molecular mechanisms. These include the formation of midline glial populations and the expression of specific molecules required to guide callosal axons as they cross the midline [17]. Congenital CC structural abnormalities include: agenesis (ACC), with either complete or partial absence of this structure; hypoplasia, referring to CC that is thinner than expected; and dysgenesis, which results when the CC is present but malformed in some way [18,19]. ACC is among the most common brain malformations observed in humans [20]. The prevalence of ACC ranges from 0.5 in 10,000 in the general population to 600 in 10,000 in children with neurodevelopmental disability [21]. The most frequent causes of ACC are gene mutations that are related to pathways of axon guidance, ciliary development, and cell adhesion, proliferation, differentiation and migration [22]. ACC is a feature of hundreds of different disorders, and all modes of inheritance have been observed [21]. Rarely, it may occur as an isolated malformation in the absence of other major abnormalities [13]. More often, ACC is a component of more complex malformation syndromes [13,23,24]. ACC can occur in association with disorders of neuronal and/or glial proliferation, neuronal migration and/or specification, midline patterning, axonal growth and/or guidance, and post-guidance development [25]. Abnormalities of the CC are noted in more than 1300 unique OMIM entries [26], suggesting that CC development is very sensitive to genetic perturbations [13]. There are more than a thousand different syndromes and metabolic disorders associated with CC disorders, most of them leading from moderate to severe neurodevelopmental disability [27]. Syndromes with ACC can be broadly classified by the stage in development that is primarily affected [12,25]. It is important to emphasize how patients with similar CC anomalies detected using MRI can present different clinical manifestations [28]. In a recently published article, in the list of gene mutations responsible for CC anomalies, *IKBKG* is not mentioned [29].

To date, there has been a lack of publications dealing directly with the impact of the *IKBKG* gene on the development and occurrence of CC abnormalities. Considering the frequency of CC anomalies in IP and the fact that the most frequent causes of ACC are gene mutations [22], it would be useful to investigate the relationship between their occurrence and gene mutations in IP patients. Using MRI and genetic analyses, the aim of this study was to determine the presence of CNS abnormalities, especially CC anomalies in IP patients; their relationship with the *IKBKG* gene mutations; the possible presence of other gene mutations; and the X-chromosome inactivation pattern.

## 2. Materials and Methods

### 2.1. Patients

The study enrolled a group of seven IP patients who had a clinically confirmed diagnosis of IP, according to the updated IP diagnostic criteria [10], and a positive finding of *IKBKG* exon 4–10 deletion.

### 2.2. Genetic Analyses

Genetic analyses of the *IKBKG* gene and the X-chromosome inactivation, analysis of the genes relevant for neurodegeneration, and whole exome sequencing (WES) analyses were performed according to the methodology reported in Minić et al. [30]. WES was performed by 3billion, Inc. Seoul, Republic of Korea to make sure that there were no additional clinically significant variants/genes missing, and the gene panel was created [30].

#### 2.2.1. Genetic Analyses of the *IKBKG* Gene

For the molecular genetic examination, we used the DNA extracted from a peripheral blood sample. The genetic analysis of each patient began by confirming the *IKBKG* exon 4–10 deletion using an improved PCR method [31,32]. In order to elucidate the patients’ phenotypes, further molecular genetic analyses were performed.

#### 2.2.2. X-Chromosome Inactivation

The study of the X-chromosome inactivation pattern was performed by the examination of the methylation status of the AR locus, as previously described in the literature [33,34]. Genomic DNA restriction was digested with enzymes *HpaII* and *RsaI*, and a PCR amplification of the selected AR locus region was performed. Products were separated using the ABI 3500 Genetic Analyzer (Life Technologies, Carlsbad, CA, USA).

#### 2.2.3. Analysis of Genes Relevant for Neurodegeneration

Additionally, “clinical exome” next-generation sequencing (NGS) was performed following the manufacturer’s instruction [35] on an Illumina MiSeq platform (Illumina, San Diego, CA, USA) using TruSight One Panel (Illumina, San Diego, CA, USA), which comprised coding the regions of 4813 genes associated with clinically relevant phenotypes. Using Illumina’s Variant Studio v.3.0, a data analysis was performed according to the phenotypic characteristics of the patients. A virtual gene panel was then created, comprising 185 genes relevant for neurodegeneration listed in Minić et al., 2022 [30]. Only the variants that passed quality filters and had a global frequency of <5% were considered. NGS analysis singled out a heterozygous variant c.1448T > G (p.L444R) in the *GBA* gene in one patient. This result was then confirmed by Sanger sequencing [36].

#### 2.2.4. Whole Exome Sequencing (WES) Analysis

As Illumina TruSight One Panel does not include all genes, WES was performed by 3billion, Inc. Seoul, Republic of Korea. Genomic DNA was extracted from whole blood using QIAamp DNA Blood Mini Kit (QIAGEN, Germantown, MD, USA). Exome capture was performed using xGen Exome Research Panel v2 (Integrated DNA Technologies, Coralville, IA, USA), and sequencing was performed using Novaseq 6000 (Illumina, San Diego, CA, USA). Sequencing data were processed as previously described [37]. Variant interpretation, including variant annotation, filtering and classification, was performed using EVIDENCE, a software program developed by 3billion [37]. Variants were classified following the ACMG guideline [38], and symptom similarity scores were calculated based on the patient’s phenotype using a scoring system developed by 3billion [37]. Variants were manually curated by 3billion’s medical team.

### 2.3. Neuroradiological Examinations

Neuroradiological examinations of all patients were performed using MRI on a 1.5 T scanner. All patients underwent T1- and T2-weighted sequences (or fluid attenuated inversion recovery). Diffusion-weighted MRI was a part of the standard MRI protocol (b factor, 1000 s/180 mm^2^). Brain MRI was analyzed by experienced neuroradiologists.

### 2.4. Inclusion Criteria

The criterion for including patients in this study was the presence of the *IKBKG* mutation and the absence of another mutation in IP patients who met the updated diagnostic criteria [10] for IP. The criterion was confirmed using WES analysis.

### 2.5. Exclusion Criteria

The criterion for excluding IP patients from further study was the presence of a mutation of any other gene except *IKBKG*.

### 2.6. Literature Data Analysis

In addition to the analysis of the examined IP patients, a short review of data was also performed from the published literature. The review concerned IP patients with observed CC anomalies using neuroimaging methods published in the literature in the period 1993–2012 [39,40,41,42,43,44,45,46,47,48,49,50,51,52,53]. These results were not present in the finally published version of the quoted manuscript [9].

## 3. Results

### 3.1. Results Concerning Patients

A group of seven female patients with IP was examined, four of which had neurological disorders. All patients were positive for *IKBKG* exon 4–10 deletion, confirmed by WES analysis. Only one patient who had *IKBKG* exon 4–10 deletion additionally had an NGS and WES analysis-detected heterozygous *GBA* mutation responsible for Gaucher disease (OMIM * 606,463, ORPHA 355). According to the exclusion criteria, this patient was excluded from further research. This patient was recently presented in a separate paper [30]. All patients were examined for neurological, ophthalmological, and dental findings and underwent MRI, and the results are presented in Table 1. Different clinically observed CNS anomalies and CNS findings were diagnosed using MRI and are presented in Table 2 and Table 3.

### 3.2. Case Reports

#### 3.2.1. Case 1

Upon first examination at the age of two, the patient presented hyperpigmented macules along Blaschko lines on the trunk and a few verrucous lesions. The first skin changes were reported to have appeared immediately after birth. A skin biopsy was performed, which confirmed IP. Early neurological examination revealed hypertonia, reduced motor activities and hyperactive deep tendon reflexes. Electroencephalographic (EEG) recording showed focal epileptic activity, but the patient did not have seizures at all. Later on, psychomotor development showed moderate delay in achievement of gross motor milestones.

**Figure 1:**
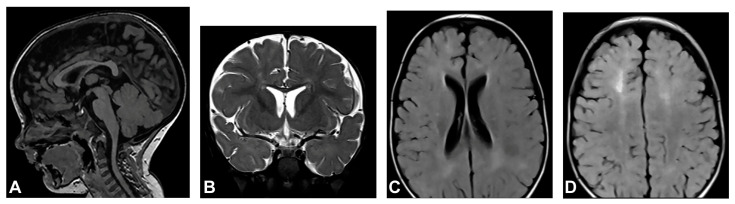
(Case 1) (**A**) Sagittal T1 MPRAGE magnetic resonance image at the age of two shows macrocrania, thinning of CC and a pineal gland cyst. (**B**) Coronal T2W magnetic resonance plane shows mild atrophy of bilateral frontal white matter with dilated extracerebral liquor spaces. (**C**,**D**) Axial T2 FLAIR magnetic resonance planes show that lateral ventricles are slightly dilated with gliotic changes in periventricular and frontal regions.

The patient started to walk unsupported from the age of two and a half with both feet in valgus position. Upon teeth eruption, the patient presented peg-like teeth. At the age of five, she had spastic quadriparesis with paraparetic gait, prominent speech delay and moderate intellectual disability. MRI of the brain showed macrocrania with bilateral loss of white matter volume in the frontal regions, dilated extracerebral liquor spaces and slightly dilated lateral ventricles (Figure 1). White matter abnormalities (gliosis) in periventricular and frontal regions were observed, as well as, hypoplasia of CC with slightly thinner body and a pineal gland cyst.

#### 3.2.2. Case 2

**Figure 2:**
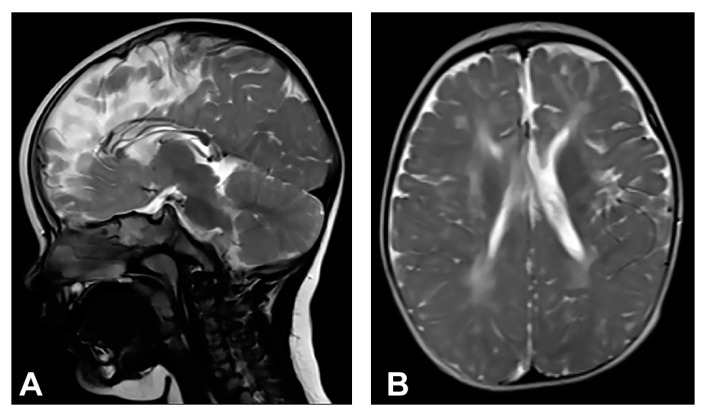
(Case 2) (**A**) Sagittal T2W magnetic resonance image at the age of one shows CC hypoplasia especially of the rostrum and body. (**B**) Axial T2W magnetic resonance image shows brain atrophy, supratentorial white matter reduction with elevated IS on T2W/FLAIR and gliotic changes. Mild bilateral non-progressive ventriculomegaly is more prominent on the left.

Skin changes were visible upon birth that evolved from stage I to stages III and IV of IP. Neonatal convulsions occurred immediately after birth. Skin biopsy was performed, and IP was histopathologically confirmed in both the patient and her mother. *IKBKG* exon 4–10 deletion was genetically confirmed in the patient’s mother as well. The patient presented dental and oral anomalies such as peg-like teeth, delayed eruption, and gothic palate. Ophthalmological examination revealed strabismus. The patient was severely delayed in terms of psychomotor development, partially due to spasticity. She started to walk independently from the age of five and a half but without a normal walking pattern. Neurological examination revealed microcephaly, spastic quadriparesis more severe on the right side, intellectual disability and prominent speech delay. Physical and speech rehabilitation were performed every day. She had focal epileptic seizures from the age of one and a half, and EEG recording showed focal epileptic activity. Antiepileptic therapy was given (valproate), and good control of seizures was achieved. MRI of the brain showed microcephaly and brain atrophy with atrophy of the thalamus and the basal ganglia (Figure 2). Supratentorial white matter loss was observed with an increased signal on T2W/FLAIR and mildly enlarged lateral ventricles, more evident on the left side. In addition, it revealed a marked hypoplasia of the CC, especially of the rostrum and body.

#### 3.2.3. Case 3

**Figure 3:**
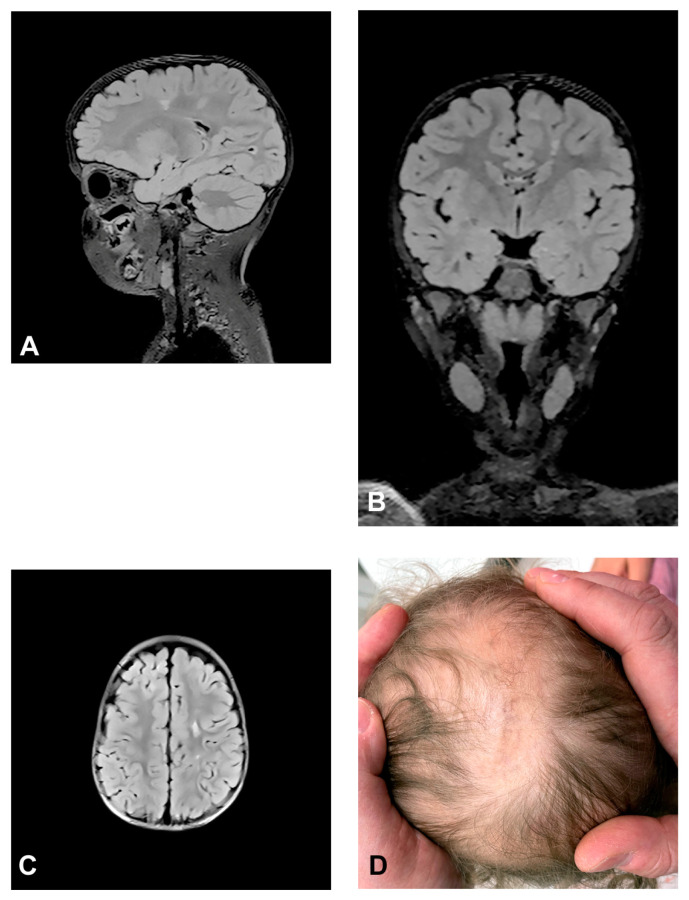
(Case 3) Sagittal (**A**), coronal (**B**) and axial (**C**) T2 FLAIR magnetic resonance images at the age of 2.5 showing small foci of hyperintensities, which appear in a band and triangle-like shape, spreading from subcortical white matter to the left lateral ventricle similarly to radial migration lines. (**D**) IP skin changes in the form of Blaschko lines on the same side of the scalp where the small foci of hyperintensities were found.

The patient presented stage I and II skin changes following Blaschko lines on the extremities and trunk upon birth. IP skin changes were on the same side of the scalp where small foci of hyperintensities were found using MRI (Figure 3). The patient presented oral anomalies typical for IP, such as gothic palate. Upon neurological examination, there were mildly decreased muscle tones with normal reflexes and motor activity. The patient had mild psychomotor delay, developing unsupported walk from the age of 19 months. She displayed mild speech delay. MRI of the brain showed small foci of hyperintensities, which appeared in a band and triangle-like shape, spreading from subcortical white matter to the left lateral ventricle.

#### 3.2.4. Case 4

**Figure 4:**
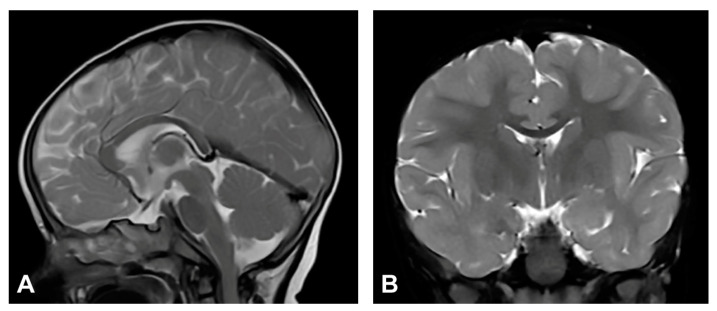
(Case 4) (**A**) Sagittal and (**B**) coronal T2W magnetic resonance images at the age of nine months without pathological findings.

The patient was one and a half months old at the initial visit, presenting hyperpigmented macules following Blaschko lines and a few verrucous papules. Two biopsies were performed depicting stage II and III of IP. After six months, skin changes were at stage III. As in previous stages, the affected area was devoid of terminal hair. The patient was diagnosed with retinopathia ischemica proliferativa. The patient also presented a gothic palate, delayed eruption and peg-like teeth. At the age of six months, the neurological examination revealed mild hypertonia, symmetrical motor activity and brisk deep tendon reflexes. The patient achieved slightly delayed developmental milestones, starting to walk without support from the age of two. Speech development was also mildly delayed. MRI showed no pathological findings (Figure 4).

#### 3.2.5. Case 5

**Figure 5:**
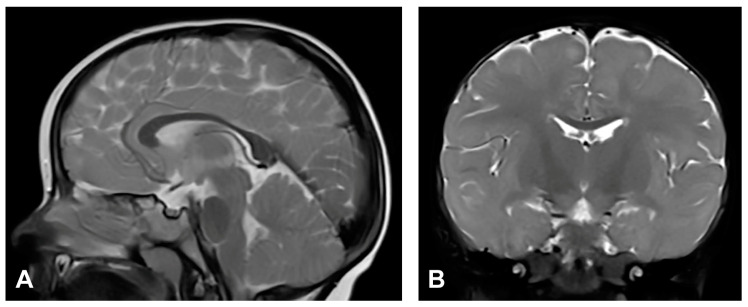
(Case 5) (**A**) Sagittal and (**B**) coronal T2W magnetic resonance images at the age of two without pathological findings.

Upon birth, the patient presented hyperpigmented and verrucous skin changes along Blaschko lines corresponding to stages II and III of IP. Histopathological analysis of skin biopsy confirmed IP. The patient also presented a gothic palate and peg-like teeth. Besides the patient, *IKBKG* exon 4–10 deletion was genetically confirmed in the patient’s mother. The patient was diagnosed with retinopathia prematuri, and anti-VEGF therapy was administered. Developmental milestones were within the physiological range and neurological development was normal. The patient could sit and walk unsupported and pick up objects with a pincer grip. EEG showed focal epileptic activity but without seizures. MRI showed no pathological findings (Figure 5).

#### 3.2.6. Case 6

**Figure 6:**
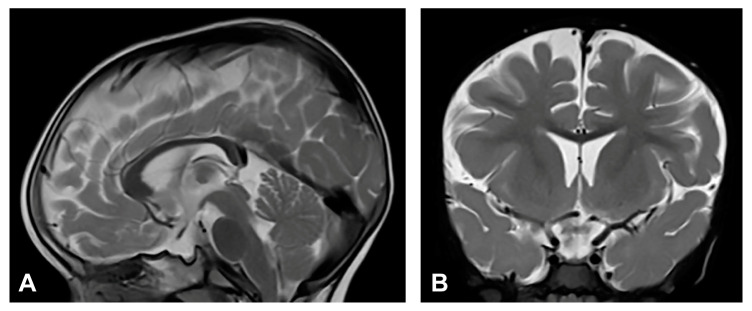
(Case 6) (**A**) Sagittal and (**B**) coronal T2W magnetic resonance images at the age of one and a half without pathological findings.

The patient was two weeks old at the initial visit and presented with vesiculobullous lesions grouped in stripe-like shapes following the lines of Blaschko. Three months later, the patient had several verrucous changes, hyperpigmented macules, and a very few vesicles. When six-months-old, there were Blaschko linear, slightly erythematous and pigmented changes forming atrophic lines with a verrucous part. Biopsy was performed, confirming IP. The patient presented stage I solitary and grouped vesicles in a linear arrangement with yellowish content and serocrusts on an erythematous skin. Skin hair was significantly reduced in the affected area. Early verrucous lesions were yellowish or whitish on an erythematous and slightly pigmented skin. The patient was ophthalmologically diagnosed with retinopathia prematuri and administered anti-VEGF therapy. The patient also had a gothic palate and peg-like teeth. Neurological development was normal. MRI revealed no pathological findings (Figure 6).

### 3.3. Literature Data Analysis

Un-summarized data published in the literature in the period 1993–2012 [9] were analyzed regarding 89 IP patients with neurological disorders who underwent CNS neuroimaging analysis. The review of the data showed that 22 females (24.7%) had CC anomalies (Table S1, Supplementary Material). A subsequent detailed analysis of these IP patients’ CNS anomalies showed that only one had hypoplasia of the CC without the presence of other CNS anomalies [9]. Of the remaining 21 patients, 18 had CC hypoplasia, all with other, associated CNS anomalies, two patients had CC agenesis, and one had a CC stroke.

## 4. Discussion

All examined patients diagnosed according to the updated diagnostic criteria [10] had skin changes characteristic of IP in different stages. All seven female IP patients had *IKBKG* exon 4–10 deletion. In addition to the *IKBKG* exon 4–10 deletion, one patient (Case 7) also had a heterozygous variant of the *GBA* gene and was excluded from further research. WES analysis showed that the remaining six IP patients had no significant mutations other than *IKBKG* exon 4–10 deletion, the most common IP-causing mutation. Four of six remaining IP patients (Cases 1–4) had clinically diagnosed CNS disorders, and one (Case 5) had EEG showing pathological focal epileptic activity. One patient (Case 6) had normal neurological development. Hypoplasia of the CC along with other brain changes (atrophy, gliosis, ventricular dilatation) was found in two patients (Cases 1 and 2) using MRI. Case 1 also had macrocrania. In the remaining three IP patients (Cases 4–6), no morphological changes of the brain were found using MRI, but they were diagnosed with severe retinopathy.

In the group of IP patients that was examined using MRI, three out of six had positive CNS findings. The MRI findings in two patients (Cases 1 and 2) in which the CC was affected were severe, and in the third (Case 3), mild.

The findings of X-chromosome inactivation in these patients were interesting. Namely, Cases 1 and 3 had a random type X-chromosome inactivation, and Case 2 had skewed X-chromosome inactivation. Furthermore, Case 3 with mild MRI findings had a random X-chromosome inactivation.

The process of X-chromosome inactivation is very complex and takes place in a certain short period of time [54] (p. 141). Due to its easy availability in comparison to other tissues, monitoring of X-chromosome inactivation is usually performed on the DNA of leukocytes. The choice of active X-chromosome is random at onset, but the mutant allele in expressing cells adversely affects their proliferation, leading to the overgrowth of the wild-type cells. Skewing can also occur when the choice of the active X-chromosome during embryogenesis is biased because of mutations that affect the choice process [54] (p. 175). In the case of IP, it is the disease process itself that causes the death of mutant cells, which explains their growth disadvantage [54] (p. 185). There is a severe mutant cell selection in IP [54] (p. 237). A significant association of the X-inactivation ratios between each tissue is present in most individuals, but wide variations were apparent in some cases, making accurate extrapolations between tissues impossible [55]. In the general population of normal females, 15% of the X-chromosome had skewed patterns of inactivation [56]. X-chromosome inactivation ratios in normal women depend on age and tissue type [55]. Results show that the incidence of severe skewing is relatively common and increases with age, occurring in 7% of women under 25 years of age and 16% of women over 60 [55]. On the other hand, such an investigated group may include normal females who are unidentified carriers of one or more deleterious alleles [54] (pp. 180–181). In the literature, there is a known case of a female patient with IP, heterozygous for a less severe *IKBKG* mutation [57]. Over a period of four years, a pattern of X-chromosome inactivation in her granulocytes and T cells that was random at birth gradually changed to one in which all of the cells expressed the normal allele, presumably because of the progressive death of mutant cells. The percentage of cells expressing the normal allele was 55% at the age of 24 months, and 100% at four years of age [57]. These findings show all the complexity of X-chromosome selection and the difficulties in their interpretation.

Dangouloff-Ross et al. [58] reviewed brain MRI findings of 18 female patients with genetically proven IP. They found five IP patients with normal MRI, six patients with mild white matter abnormalities with cortical and CC atrophy, and seven patients with severe cortical abnormalities suggesting a vascular disease. Most patients with severe abnormalities had random X-inactivation (6/7, 86%), while 80% (4/5) of patients with normal MRI and 100% (6/6) of patients with mild white matter abnormalities had skewed inactivation. They concluded that skewed X-chromosome inactivation may protect the brain from damage, while in the case of random inactivation, the expression of the mutated *IKBKG* gene may lead to severe brain lesions. Based on the findings of Danguloff-Ross et al. [35], one would expect both Cases 1 and 2 presented in this study to have random X-chromosome inactivation, but Case 2 had skewed X-chromosome inactivation. Skewed X-chromosome inactivation in Case 2 did not prevent severeness of IP. The occurrence of skewed X-chromosome inactivation in Case 2 and random X-chromosome inactivation in Case 3 can be explained in several ways. It is possible that the results for the investigated patients were influenced by the age of the patients [55] at the time of blood sampling. As even in the normal population of women, there is a small percentage of skewed X-chromosome inactivation pattern [56], this may have been the case in Case 2.

Dental and oral anomalies were found in all six examined IP patients. Two patients had delayed teeth eruption, four patients had peg-like teeth, and five patients had a gothic palate. These findings are consistent with dental and oral findings in other patients with IP [59].

MRI shows brain changes in most patients with neurological disease associated with IP [60]. In IP patients without neurological disease, MRI does not reveal abnormalities [60]. There is also no correlation between the size of the affected skin surface and the severity of the CNS changes [61]. In general, an IP patient can have a large area of the skin affected by changes, but not have MRI-detected CNS disorders, and conversely, the affected skin area can be small, and the MRI-detected CNS changes very pronounced [61]. Abnormalities are located in the cerebral hemisphere contralateral to the most severely affected side of the body [60], as documented in Case 2. The most severe MRI changes are subjacent to the scalp areas where the most severe cutaneous lesions appear located in the neonatal period [60]. In the presented Case 3, a combination of typical IP skin changes on the scalp and underlying gliotic changes of the brain detected by MRI was observed. This phenomenon was first described by Pascual-Castroviejo et al. 1994 [62]. In the available literature, there is no explanation of this phenomenon except a suggestion that cutaneous and subjacent brain lesions have the same pathogenesis during the neonatal period [60]. There is a possibility that this happened during early embryogenesis, during the embryonic disc stage, when the neural plate was formed and the ectoderm and neuroectoderm separated [15] (pp. 79–80). If at their very border there was a group of mutated cells, one part of which entered the nerve plate and the other part into the ectoderm, it is possible that during development, because of cell migrations, they found themselves opposite each other, one in the skin, and the other in the CNS. However, there is no evidence to support this hypothesis.

In IP, the most frequent clinically diagnosed CNS types of anomalies were seizures, motor impairment, mental retardation, and microcephaly. The most frequently registered CNS lesions found using imaging methods were brain infarcts or necrosis, brain atrophies, and CC lesions [9]. Apart from these, a white matter hyperintensity T2/FLAIR signal was common [63].

In the group of examined patients, two (Case 1 and Case 2) had severe CNS disorders on MRI. Case 1 had macrocrania, bilateral loss of white matter, dilated extracerebral liquor spaces and slightly dilated lateral ventricles, and gliosis in periventricular and frontal regions. Case 2 had microcephaly and brain atrophy with atrophy of the thalamus and the basal ganglia, supratentorial white matter loss with an increased signal on T2W/FLAIR and mildly enlarged lateral ventricles. Both patients had significant CC changes. Case 1 had hypoplasia of the CC, with a slightly thinner body, and Case 2 had marked hypoplasia of the CC, especially of the rostrum and body. According to the majority of authors, the hypoplasia of the CC is not a primary part of the disease, but is secondary to the cortical and subcortical neuronal lesions in the cerebral hemispheres [11,60,62]. Axons are principal constituents of the CC. Their loss occurs due to neuronal death in the cerebral cortex [14]. CNS lesions in IP may arise from the same mechanism as in the skin, by inducing apoptosis in *IKBKG*-mutated neurons [9,61]. Sarnat [14] suggested that chronic brain ischemia may lead to atrophy of the corpus callosum. Although some studies have reported ischemic changes involving distinct vascular territories, in most cases lesions affect the microvasculature [63]. Bodemer [64] supposed that the primary cause of the central nervous system lesions is, therefore, vasculopathy with the possibility of a secondary inflammation process. Minić et al. [9] suggested that apoptosis could be triggered by the *IKBKG* mutation in affected cells in blood vessel walls, as it occurs in other mutation-affected tissues, resulting in compromised vascularization, leading to brain infarction with ischaemic and hemorrhagic necrosis. Considering that, in CNS, pericytes have the same neuroectodermal origin as nerve cells [65], it is possible that the *IKBKG* mutation causes apoptosis in them as well as in neurons. Taking into account the concept of the neurovascular unit and the relationship between brain cells and their blood vessel cells, the embryonic origin of pericytes, and the pericytes’ high susceptibility to oxidative stress, it is possible that oxidative stress occurs in IP patients’ pericytes [66]. However, data on the findings of oxidative stress in IP are very rare; only one paper was found on PubMed [67] in which it was analyzed, so the possible role of oxidative stress in IP can only be speculated for now. In Case 1, a pineal cyst was found. Pineal cysts are common, usually asymptomatic, and typically found incidentally [68].

ACC is the most common major cerebral malformation that is neither lethal nor produces major neurological disabilities in all cases [14]. Complex forms of ACC are those with associated brain findings and/or syndromic, chromosomal, or genetic conditions [24]. These associated conditions are usually associated with a significantly worse prognosis [24]. In two investigated IP patients with ACC (2/2), as well as in corresponding IP patients from the analyzed literature in the period 1993–2012 (21/22) (Supplementary Material S1), ACC was usually associated with other CNS disorders that contributed to a more severe clinical picture. This was also the case with the two examined patients. Case 1 had hypoplasia of the CC with a slightly thinner body, while Case 2 had a marked hypoplasia of the CC, especially the rostrum and body. The rostrum or splenium of the corpus callosum may be preferentially affected [14].

In Case 3, brain MRI showed small foci of hyperintensities, in the form of a band and triangle-like shape similar to radial migration lines, spreading from subcortical white matter to the left lateral ventricle. It can be assumed that the presented lines were Blaschko line analogies in CNS, similar to those in the skin, representing the trace of development of the clone of neurons arising from the cell marked with the *IKBKG* mutation [69].

IP ocular anomalies were more frequent than CNS anomalies, at 37.24% and 30.44%, respectively [10]. Ophthalmological examinations showed changes in the eyes in three investigated IP patients. Case 4 was diagnosed as retinopathia ischemica proliferativa, and Cases 5 and 6 as retinopathia prematuri. In Cases 5 and 6, anti-VEGF therapy was administered. Retinopathies represent serious vision-threatening anomalies and may lead to vision-threatening manifestations or even blindness caused by retinal disease [10]. It is assumed that the pathogenesis of retinopathy in IP is similar to the pathogenesis of CNS changes, that is, that the apoptosis of vascular cells with *IKBKG* mutation is its basis [9].

All things considered, the results and findings obtained from the examinations in our research are in accordance with the expected findings in patients with IP [10]. The application of WES made it possible to demonstrate the presence of the *IKBKG* mutation and the absence of other gene mutations that could cause CNS changes in the examined IP patients. Such was a previously presented Case 7 [30] with severe IP, which, in addition to the *IKBKG* mutation, had another mutation, *GBA*, and was, therefore, excluded from further research.

## 5. Conclusions

The presented clinical and imaging findings and analyzed data from the literature suggest that all changes in the CNS basically originate from *IKBKG* mutations in neurons or in the vascular cells of its blood vessels. The simultaneous presence of *IKBKG* exon 4–10 deletion and CC abnormalities in IP patients, and the absence of other mutations demonstrated by WES analysis, indicates that *IKBKG* gene mutation is the cause of CC abnormalities and other CNS abnormalities and should be included in the list of genes responsible for CC abnormalities.

## Figures and Tables

**Table 1:** Facts concerning basic patient data and key clinical findings.

Patient	Age of Onset	Sex	IP Diagnostic Criteria Positive	*IKBKG* Exon 4–10 Deletion Positive	Additional Genetic Findings Other than *IKBKG* Exon 4–10 Deletion	X-Chromosome Inactivation Pattern	Skin Stages	CNS Anomalies	Eye Anomalies	Dental and Oral Anomalies
1	Upon birth	F	Yes	Yes	No	Random (60/40%)	II, III	Yes	No	Yes
2	Upon birth	F	Yes	Yes	No	Skewed (85/15%)	III, IV	Yes	No	Yes
3	Upon birth	F	Yes	Yes	No	Random (50/50%)	I, II	Yes	No	Yes
4	Upon birth	F	Yes	Yes	No	Skewed (90/10%)	II	No	Yes	Yes
5	Upon birth	F	Yes	Yes	No	Random (55/45%)	I, III	No	Yes	Yes
6	Upon birth	F	Yes	Yes	No	Skewed (85/15%)	II, III	No	Yes	Yes
7 (excluded from further research because of additional heterozygous *GBA* mutation)	Upon birth	F	Yes	Yes	Yes Heterozygous variant c.1448T > G (p.L444R) in *GBA* gene	Random (55/45%)	III	Yes	Yes	Yes

Legend: F-female, IP-Incontinentia pigmenti, WES-Whole Exome Sequencing, CNS-Central Nervous System, MRI-Magnetic Resonance Imaging.

**Table 2:** Different clinically diagnosed CNS anomalies according to clinical findings.

Patient	Age at the Time of Onset of Neurological Disorders and Current Age	Seizures	Pathological EEG	Motor Impairment	Mental Retardation	Microcephaly	Rarely Diagnosed or Unspecified CNS Anomalies
1	First year of life Now aged 5 years	No	Yes	Yes	Yes	No	Yes
2	Neonatal period Now aged 8.5 years	Yes	Yes	Yes	Yes	Yes	No
3	Neonatal period Now aged 2.5 years	No	No	Yes	Yes	No	No
4	First year of life Now aged 3 years	No	No	Yes	No	No	No
5	Neonatal period Now aged 4 years 3 months	No	Yes	No	No	No	No
6	Neonatal period Now aged 2.5 years	No	No	No	No	No	No

Legend: CNS-Central Nervous System, EEG-electroencephalogram.

**Table 3:** Different CNS findings diagnosed using MRI.

Patient	Brain Infarction	White Matter Abnormalities	Brain Atrophies	Brain Ventricular Dilatation	CC Anomalies	Brain Cystic Lesions	Rarely Diagnosed or Unspecified CNS Findings
1	No	Yes	Yes	Yes	Yes	No	Yes
2	No	Yes	Yes	Yes	Yes	No	No
3	No	No	No	No	No	No	Yes
4	Normal neuroimaging findings	No	No	No	No	No	No
5	Normal neuroimaging findings	No	No	No	No	No	No
6	Normal neuroimaging findings	No	No	No	No	No	No

Legend: CNS-Central Nervous System, MRI-Magnetic Resonance Imaging, CC-corpus callosum.

## Data Availability

Data are contained within the article and the Supplementary Materials.

## Supplementary Materials

The following supporting information can be downloaded at: https://www.mdpi.com/article/10.3390/diagnostics13071300/s1, Table S1: Distribution of CC abnormalities diagnosed using brain imaging in IP patients with CNS lesions for the period 1993–2012 (unpublished data taken from study Minić et al., 2013).
