# Supplementary material for: The Impact of the IKBKG Gene on the Appearance of the Corpus Callosum Abnormalities in Incontinentia Pigmenti
Source: Diagnostics (Basel). 2023 Mar 30;13(7):1300. doi: 10.3390/diagnostics13071300 (PMC10093331; doi:10.3390/diagnostics13071300)
Supplement: Supplementary file 1 [file diagnostics-13-01300-s001.zip › diagnostics-2267410-supplementary.pdf]

The Impact of the *IKBKG* Gene on the Appearance of the Corpus Callosum Abnormalities in Incontinentia Pigmenti

Snežana Minić [1,*], Nataša Cerovac [2], Ivana Novaković [3], Slobodan Gazikalović [4], Svetlana Popadić [1] and Dušan Trpinac [5]

Supplementary material

Table S1. Distribution of CC abnormalities diagnosed using brain imaging in IP patients with CNS lesions for the period 1993-2012 [unpublished data taken from study Minić et al., 2013]

Number	Reference	CC hypoplasia	CC agenesis	Associated CNS abnormalities
1	Mangano and Barbagallo, 1993	Yes		Low-density areas next to lateral ventricle, and in white matter of parietal region, a mildly enlarged lateral ventricles
2	Albernaz et al, 1997		Yes	Bilateral anophthalmos, an absence of the optic chiasm, the optic nerves are very small, right lateral ventricle is dilated, hippocampal hypoplasia, the posterior white matter tracts were also hypoplastic
3	Aydingöz and Midia, 1998	Yes		Enlargement of the left lateral ventricle, periventricular white matter lesions
4	Aydingöz and Midia, 1998	Yes		Symmetrical enlargement of the lateral ventricles, periventricular white matter lesions
5	Vicente-Villa et al 2001	Yes		Cystic lesions in white matter, subcortical occipital bilateral gliosis
6	Hadj-Rabia et al, 2003	Yes		Cerebral atrophy, porencephalia, hemorrhagic necrose
7	Hadj-Rabia et al, 2003	Yes		No
8	Boor et al, 2004	Yes		Cerebral atrophy, enlarged ventricles
9	Pascual-Castroviejo et al, 2006	Yes		White matter and cortical gray matter lesion in left hemisphere
10	Pascual-Castroviejo et al, 2006	Yes		White matter and cortical gray matter lesion in left hemisphere, right cerebellar hemisphere lesion
11	Pascual-Castroviejo et al, 2006	Yes		White matter and cortical gray matter lesion in left brain hemisphere
12	Pascual-Castroviejo et al, 2006	Yes		Bilateral white matter and cortical gray matter lesion
13	Pascual-Castroviejo et al, 2006	Yes		Bilateral white matter and cortical gray matter lesions
14	Maingay-de Groof et al, 2008	Yes		Atrophy of cerebellar vermis, gliosis
15	Lee et al, 2008	Yes		Multiple dot-like lesions with low signal intensity in the corona radiata of both cerebral hemispheres, periventricular white matter lesions

16	Emre et al, 2009	Yes		Encephalomalacic alterations in the left frontal lobe, enlargement of adjacent subarachnoid spaces
17	Cartwright et al, 2009	CC stroke		Multiple areas of restricted diffusion throughout both brain hemispheres, multiple, bilateral acute strokes, bilateral frontal and occipital cystic encephalomalacia due to the prior infarcts
18	Demirel et al, 2009		Yes	Midline skull defect and an associated lesion protruding to the subcutaneous tissue consistent with encephalocele, an arachnoid cyst in the posterior fossa, a porencephalic cyst in the left parietal lobe
19	Al-Zuhaibi et al, 2009	Yes		Atrophy of the frontal horns, abnormality in the periventricular white matter associated with brain atrophy
20	Abe et al, 2011	Yes		Changes in subcortical white matter, deep white matter, basal ganglia, amd thalamus, cystic encephalomalacia with atrophic changes in the right frontal area
21	Abe et al, 2011	Yes		Changes in subcortical white matter, deep white matter, basal ganglia,and thalamus. Acute stage of encephalopathy
22	Fryssira et al, 2011	Yes		Porencephalic cyst on the left frontal lobe atrophy of the corpus callosum, loss of white matter bilaterally
